# Hereditary Ataxias: From Bench to Clinic, Where Do We Stand?

**DOI:** 10.3390/cells13040319

**Published:** 2024-02-09

**Authors:** Federica Pilotto, Andrea Del Bondio, Hélène Puccio

**Affiliations:** Institut Neuromyogène, Pathophysiology and Genetics of Neuron and Muscle, Inserm U1315, CNRS-Université Claude Bernard Lyon 1 UMR5261, 69008 Lyon, France

**Keywords:** ataxia, cerebellum, therapy

## Abstract

Cerebellar ataxias are a wide heterogeneous group of movement disorders. Within this broad umbrella of diseases, there are both genetics and sporadic forms. The clinical presentation of these conditions can exhibit a diverse range of symptoms across different age groups, spanning from pure cerebellar manifestations to sensory ataxia and multisystemic diseases. Over the last few decades, advancements in our understanding of genetics and molecular pathophysiology related to both dominant and recessive ataxias have propelled the field forward, paving the way for innovative therapeutic strategies aimed at preventing and arresting the progression of these diseases. Nevertheless, the rarity of certain forms of ataxia continues to pose challenges, leading to limited insights into the etiology of the disease and the identification of target pathways. Additionally, the lack of suitable models hampers efforts to comprehensively understand the molecular foundations of disease’s pathophysiology and test novel therapeutic interventions. In the following review, we describe the epidemiology, symptomatology, and pathological progression of hereditary ataxia, including both the prevalent and less common forms of these diseases. Furthermore, we illustrate the diverse molecular pathways and therapeutic approaches currently undergoing investigation in both pre-clinical studies and clinical trials. Finally, we address the existing and anticipated challenges within this field, encompassing both basic research and clinical endeavors.

## 1. Introduction

The term ataxia derives from Greek, *a-* without and *taxis-* order, and it refers to a poor coordination of movements. Hereditary ataxias (HAs) are a group of heterogeneous neurodegenerative diseases (NDDs) that involve the degeneration of cerebellar- and extracerebellar-related circuits, leading to the development of progressive abnormal gait, dysarthria, and ataxia. Within this large family of movement disorders, other structures involved in balance and gait control might also be affected: for instance, some forms of autosomal-recessive cerebellar ataxias involve the degeneration of proprioceptive neurons located in the dorsal root ganglia (DRG) and the loss of function in muscle spindles leading to the loss of proprioception [1,2].

**Figure 1:**
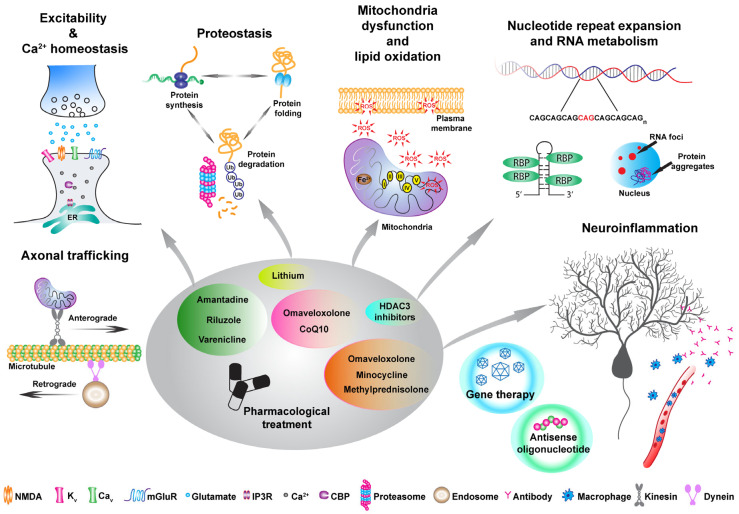
Pathophysiological changes in hereditary ataxia (HA) and the corresponding treatments. This diagram depicts various pathophysiological alterations in ataxia and suggests treatments that address specific pathways. The proposed pharmacological strategies aim to regulate excitability and calcium homeostasis, (i.e., Riluzole, Amantadine, and Varenicline) as well as target proteostasis, mitochondrial dysfunction, and inflammation, with the recently FDA-approved drug Omaveloxolone. Additionally, alternative therapeutic methods like antisense oligonucleotides and gene therapy can address distinct pathways within the proposed framework.

Given the broad spectrum of symptoms and phenotypic heterogeneity that characterize HAs, their classification is challenging. A rather conservative classification can be based on the mode of inheritance or by distinguishing between autosomal-dominant cerebellar ataxias (ADCAs), mainly known as spinocerebellar ataxia (SCA), autosomal-recessive cerebellar ataxias (ARCAs), and X-linked ataxias [2]. For example, among the ADCA category, SCA3 is the most common, together with SCA1, 2, 6, 7, and 17, which are all caused by a CAG trinucleotide expansion leading to a polyQ expansion in the encoded protein [3]. Friedreich’s ataxia (FA) is one of the most common ARCAs caused by an instable GAA expansion, followed by autosomal-recessive spastic ataxia of Charlevoix-Saguenay (ARSACS), ataxia–telangiectasia (AT), and, more recently, cerebellar ataxia with neuropathy and vestibular areflexia syndrome (CANVAS) [4,5,6]. Among the X-linked ataxias, fragile X-associated tremor/ataxia syndrome (FXTAS), caused by a CGG repeat expansion in the premutation range in the 5′ non-coding region of the fragile X messenger ribonucleoprotein 1 (*FMR1*) gene is the most common [7]. Finally, there are episodic ataxias (EAs), mostly inherited in an autosomal-dominant manner, of which the most common are EA1 and EA2, associated with mutations in the *KCNA1* potassium channel and *CACNA1A* calcium channel, respectively [8,9]. The classification presented above is a traditional approach to describing ataxias. However, for the purpose of this review, we suggest a classification centered on gene function and the pathways impacted by various mutations, as these factors may hold greater significance in terms of treatment considerations (Figure 1). Indeed, different types of ataxias display impairment in neuronal excitability and calcium homeostasis [10]: among these are SCA1 [11,12] as well as EA1 and EA2, as these mutations are directly impinging onto channels which are involved in excitability and calcium homeostasis. Different mutations such as the STUB1 found in patients with ARCA are involved in the ubiquitin proteasomes systems, therefore impairing proteostasis [13]. Moreover, polyQ-expanded ATXN3 has been found to be involved in the autophagy pathway [14]. Mitochondrial dysfunction also seems to be an appealing candidate for future therapeutical interventions. Indeed, oxidative stress, lipid oxidation, mitochondria dysfunction, and ATP production deficits are common denominators of different HAs such as FA, ARSACS, ARCA2, SCA1, SCA3, and SCA7, to list a few [15,16,17,18,19,20]. Finally, many HAs, including SCA1, 2, 3, 36, and many others, are characterized by nucleotide repeat expansion, which might alter RNA metabolisms leading to RNA toxicity via the sequestration of RNA-binding proteins and RNA foci formation within vulnerable neurons [21,22,23,24].

Given the broad spectrum of symptoms, the different genetic pattern of inheritance, and the various molecular pathways implicated, finding a resolutive treatment remains challenging. Nonetheless, during the past decade, encouraging advances in terms of basic as well clinical research have been achieved. In this review, we provide further insights in the field of HAs, from autosomal-dominant to X-linked ataxias, and discuss the major achievements in terms of promising pre-clinical models and therapeutical approaches. Finally, we touch upon the main challenges and limiting factors that represent an obstacle to the achievement of an effective treatment for these conditions.

## 2. Autosomal-Dominant Hereditary Ataxias

ADCAs are an heterogenous group of genetic NDDs that are numbered chronologically in the order of discovery of the genetic loci. All SCAs share common features including cerebellar dysfunctions, leading to progressive motor incoordination, dysarthria, unsteady gait, and ataxia, commonly accompanied by pyramidal and extrapyramidal tracts’ impairment [25]. In some forms, non-motor symptoms such as cognitive and retinal degeneration are also present [25]. ADCAs are classified based on their clinical manifestation in type I, II, and III. Typically, type I ADCAs (SCA1, 2, 3, 4, 8, 10, 12, 23, 25, 27, 28, 32, and 36) present clinical manifestations that involve cerebellar symptoms accompanied by different neurological manifestations such as cognitive impairment and seizure; type II ADCAs (SCA7) also present retinal degeneration, while type III ADCAs (SCA5, 6, 11, 26, 30, 31) display pure cerebellar symptomatology [26,27].

The prevalence of ADCAs is approximately between 1 and 5:100,000; however, it should be noted that there is great variability based on geographical and ethnical groups, with an incidence of 3:100,000 in the European population. Symptoms usually arise during the 4th decade of life, mainly with the progressive loss of motor coordination, mostly due to the degeneration of cerebellar Purkinje cells and the brainstem. To date, more than 49 SCAs have been classified based on 37 gene mutations that include repeat expansions in both coding and non-coding gene regions, point mutations, and deletions [2]. 

A CAG trinucleotide repeat expansion in different genes is the principal cause of the most common SCAs, including SCA1, 2, 3, 6, 7, and 17, leading to an abnormal polyQ tract within the encoded protein. There is an inverse correlation between the size of the repeat and the age of onset [28]. Furthermore, due to the genetic instability of CAG expansion, with a tendency to expand further when transmitted to the next generation, a phenomenon named anticipation, that is, the tendency for disease to worsen from generation to generation, is present [28,29,30]. Moreover, the encoded proteins arising from gene mutations play roles in various physiological processes, such as transcription regulation, proteostasis, excitability, mitochondrial metabolism, and calcium homeostasis. These collectively contribute to the development of diseases and neuronal degeneration. In the following paragraphs, we describe the most common SCAs with their clinical and neuropathological features.

### 2.1. SCA1

SCA1 (OMIM # 164400) is characterized by ataxia, dysarthria, and eventual deterioration of bulbar functions with, typically, onset in the 3rd–4th decade of life. One third of patients with SCA1 also present cognitive impairment [31,32]. Respiratory failure is the main cause of death [33]. Patients with SCA1 usually present a loss of Purkinje neurons (PN) and deep cerebellar nuclei (DCN) neurons located in the dentate nucleus, leading to olivopontocerebellar atrophy. Within the spinal cord, the anterior horns, Clarke’s column, and the spinocerebellar tract are mainly affected [3,34,35,36]. 

SCA1 is caused by a CAG trinucleotide expansion in the coding region of the *ATXN1* gene, encoding the ataxin-1 protein [37]. The ataxin-1 protein is widely expressed throughout the central nervous system, and it shuttles between the cytoplasm and the nucleus via its nuclear localization signal (NLS) domain. Ataxin-1 is involved in RNA metabolism [38] and can act as a transcriptional repressor together with the co-repressor capicua (CIC). The pathological polyQ expansion leads to the retention of the protein in the nuclear compartment, leading to its aggregation and possible toxicity. Interestingly, a single amino acid substitution in the NLS sequence is able to abolish its pathogenicity [38,39]. In physiological conditions, the ATXN1 protein forms a complex with the transcriptional repressor capicua (CIC), thereby directly modulating its activity. The gain of function of this complex can lead to PN degeneration [40,41]. Finally, phosphorylation defects in different residues of the ataxin-1 protein such as Serine 776 (Ser776) in the C-terminal domain are implicated in disease pathophysiology as they allow the interaction with different binding partners, including splicing factors [42,43,44,45]. For instance, Ser776 phosphorylation increases the interaction between Tip60 and ataxin-1, leading to the disruption of RORα-mediated gene expression and enhanced pathogenesis [46], as RORα is fundamental in the development of PNs during the late embryonic stage.

### 2.2. SCA2

Patients with SCA2 (OMIM # 183090) manifest a broad spectrum of symptoms including cerebellar ataxia accompanied by oculomotor impairment and parkinsonism due to the degeneration of dopamine-releasing neurons in the substantia nigra [47,48]. In addition, some degree of cognitive impairment has been observed in patients associated with sleep disturbances, together with either clinical or subclinical peripheral neuropathy [49,50]. Analogous to SCA1, patients with SCA2 present the same pattern of degeneration within the olivopontocerebellar structure; however, the deep cerebellar nuclei structures remain relatively spared by the pathology [51]. SCA2 is caused by a CAG trinucleotide expansion in the coding region of the *ATXN2* gene, encoding the ataxin-2 protein [47]. Unaffected individuals have 13 to 31 CAG repeats, whereas affected individuals have over 37 repeats [52]. Interestingly, intermediate CAG expansions (32–33 CAG) within the normal range are a risk factor for amyotrophic later sclerosis (ALS) and a disease modifier for patients with fronto-temporal dementia (FTD), both familial and sporadic cases [53,54,55,56]. The ataxin-2 protein is implicated in RNA metabolism by interacting with polyA-binding protein (PABP) as well as transactive response (TAR) DNA-binding protein 43 (TDP43), which also bind to RNA, suggesting an interplay of these proteins within common pathways underlying both SCA2 and ALS. This probably reflects a common risk factor for both SCA2 and ALS pathophysiology [57,58,59].

### 2.3. SCA3 (Machado–Joseph Disease)

SCA3 (OMIM # 109150) is considered one of the most common forms of SCA; nevertheless, there is great variance in its prevalence between different countries, which can span from nearly 0% to more than 55% of individuals with an ADCA in countries such as Portugal [60,61]. The onset of this pathology can vary from a very young age to adulthood, which reflects different patterns of disease progression; therefore, SCA3 can be classified based on clinical observations into four subtypes [36,62,63,64]. SCA3 type I involves patients with early onset between the age of 10 and 30 years and is characterized by nominal ataxia with the involvement of pyramidal and extrapyramidal signs. Type II, the most common, appears between the second and fifth decade of life with patients developing progressive ataxia accompanied by pyramidal signs, while type III appears later in life and mainly involves peripheral neuropathy which leads to muscle atrophy. Finally, type IV occurs at different ages, and patients experience a strong parkinsonism component. Differently from SCA1 and SCA2, the cerebellar cortex is relatively spared, but degeneration occurs in the deep cerebellar nuclei, in particular the dentate nucleus [65,66]. Moreover, degeneration of the cerebral cortex and the substantia nigra, leading to parkinsonism, as well as other subthalamic nuclei is observed in some patients [36,67]. Furthermore, patients suffer of mild cognitive impairment [68]. The unstable CAG repeat that characterizes SCA3 leads to a polyQ expansion in the encoded ataxin-3 protein. The normal range is up to 44 repeats, while SCA3 patients have between 52 and 86 repeats. Under physiological conditions, ataxin-3 shuttles between the nucleus and the cytoplasm and is a deubiquitinating enzyme involved in protein quality control regulation [69,70,71]. In SCA3, mutant ataxin-3 accumulates in the nucleus of neurons [72].

### 2.4. SCA6 

SCA6 (OMIM # 183086) is considered a pure form of cerebellar ataxia where cerebellar PNs of the cerebellar cortex are affected while other brain regions are relatively spared by the pathology compared to other SCAs [73]. On average, symptoms’ onset appears between the 4th and 5th decade of life and includes progressive imbalance and cerebellar ataxia accompanied by dysarthria and nystagmus [74,75]. Moreover, in some patients, basal ganglia dysfunction has been reported [74,75]. The highest prevalence is reported in Japan [76], whereas it is quite low in Europe and North America. CAG pathological expansion, which ranges from 20 to 33 repeats, affects the *CACNA1A* gene [77,78,79]. The *CACNA1A* gene encodes for the α1A subunit of the P/Q-type voltage-gated calcium channel Cav2.1, which is particularly abundant in the vulnerable PNs [75]. In addition to full-length CACNA1A, a shorter form generated through the use of an internal ribosomal entry site encodes for transcription factor α1ACT, whose physiological function is unclear. The polyQ repeat at the C-terminal domain of α1ACT affects PN development [80].

### 2.5. SCA7

Patients with SCA7 (OMIM # 164500) experience cerebellar ataxia, dysarthria, and spasticity [81]. A peculiar characteristic of SCA7 is retinal degeneration, leading, eventually, to blindness [82]. A small percentage of patients manifest cognitive impairment and psychosis [83,84]. Patients with adult-onset SCA7 harbor between 37 and 50 repeats, whereas non-pathological repeats are found to be between 4 and 17 CAG [81]. As for other types of SCAs, juvenile forms of SCA7 are more aggressive in terms of symptomatology and prognosis. Adolescent SCA7 is characterized by extensive visual loss at first involving macular degeneration, without initial cerebellar ataxia symptoms [81]. Infantile SCA7, which manifests at birth or within the few first months, is caused by extremely long expansions reaching 460 repeats [85], characterized by congenital malformations resulting in multiorgan failure [81]. SCA7 is characterized by a CAG expansion in the ataxin-7 gene [86]. *ATXN7* is a core component of the Spt-Ada-Gcn5 acetyltransferase (SAGA) complex that functions as a chromatin-modifying transcriptional coactivator complex [87]. Ataxin-7 directly interacts with GCN5, the histone acetyltransferase component of the STAGA/SPT3 complex, mediating the interaction with the cone-rod homeobox (CRX) transactivator of photoreceptor genes [88]. Finally, the ataxin-7 protein shuttles between the nucleus and the cytoplasm, and when it localizes itself in the cytoplasm, it has been shown to be involved in microtubule organization [89].

### 2.6. SCA17

To date, less than 100 families have been reported to be affected by SCA17 (OMIM # 607136) worldwide. Symptoms’ onset can vary from infancy to 75 years [90] and include cerebellar ataxia, pyramidal signs as well as psychiatric manifestation and dementia [91], caused by the degeneration of the cerebral cortex and cingulate and hippocampal gyri as well as cerebellar PN and substantia nigra degeneration [36]. SCA 17, also called Huntington’s disease-like-4 because of the similarity in its symptomatology with Huntington’s disease (HD), is caused by a CAG expansion in the coding region of the TATA box-binding protein (*TBP*) gene [92]. The polyQ stretches can vary in size causing different degrees of penetrance, with normal alleles containing 25 to 40 trinucleotide repeats expansions, whereas expansions containing more than 50 trinucleotides repeats are considered pathological with full penetrance [93]. TBP is an important factor for the initiation complex of RNA polymerase II, which is essential for the initiation of transcription [30]. In SCA17, polyQ expansion affects multiple cellular processes, from the Notch signaling pathway to the ER stress response, by impairing different transcription factors via gain or loss-of-function mechanisms [94]. 

### 2.7. SCA27B (GAA-FGF14 Ataxia)

Among autosomal-dominant SCAs associated with non-coding microsatellite pathological expansions, a heterozygous GAA trinucleotide expansion within the intron 1 of the fibroblast growth factor 14 (*FGF14*) gene has been identified in patients with late-onset spinocerebellar ataxia (SCA27B—OMIM #620174) [95]. The expansion was firstly identified in European and French-Canadian families, after long-range genome sequencing in families with unidentified HA. GAA expansion >250 within the *FGF14* gene accounts for 10 to 61% of unsolved ataxia cases, in different cohorts analyzed in Australia, Europe, and India [95]. Patient symptoms mainly manifest as cerebellar ataxia accompanied by downbeat nystagmus in 42% of the patients [95] and appear during the sixth decade of life. Nevertheless, there might be a possibility of an earlier onset of GAA-FGF14 ataxia [95]. Diagnosis of this form of ataxia remains challenging because of the long repeat expansion, which can go up to 300 already in healthy individuals, likely interrupted GAA repeats [95]. Nevertheless, Bonnet and colleagues have recently developed a new approach that combined long-range PCR, bidirectional repeat-primed PCRs, and Sanger sequencing for the detection of GAA-FGF14 expansions [96].

The post-mortem analysis of two French-Canadian patients showed marked vermis atrophy and PN loss accompanied by astrogliosis. Finally, in both post-mortem tissue and iPSCs-derived motoneurons, the *FGF14* transcript and protein level were found to be significantly reduced compared to the controls, suggesting that GAA expansion might lead to a reduction in *FGF14* transcription, similarly to Friedreich’s Ataxia [95]. Moreover, *Fgf14* knock-out mice have been found to display ataxia phenotype [97]. FGF14 is particularly abundant in the cerebellum and regulates the rhythmic firing property of the PN by regulating the voltage-gated sodium channels that are found in the initial axon segment [98,99]. Therefore, GAA-FGF14 might also be considered a channelopathy. 

## 3. Autosomal-Recessive Cerebellar Ataxias

Autosomal-recessive cerebellar ataxias (ARCAs) are a group of rare diseases with clinical and genetic heterogeneity. ARCAs’ clinical spectrum is heterogeneous and complex (often multisystemic), and the symptoms usually manifest during childhood or adolescence with a severe impact on the daily lives of patients [100]. ARCAs are characterized by the abnormal development and progressive neurodegeneration of the cerebellum and/or its associated afferents tracts, often accompanied by damage in other neurological (e.g., corticospinal tract, basal ganglia, peripheral nerve) or non-neurological systems (e.g., heart, muscles, pancreas) [101,102]. To date, 92 genetic diseases with autosomal-recessive inheritance show ataxia as the main clinical feature or combine ataxia with other predominant movement disorders [103]. However, due to genetic pleiotropy, this number could be even higher. In fact, genes linked to autosomal-dominant ataxia have also recently been found to cause autosomal-recessive ataxia in cases of biallelic inheritance (e.g., *AFG3L2*/SPAX5 [104], *SPTBN2*/SPARCA1 [105], *ITPR1*/Gillespie Syndrome [106], and *OPA1*/Syndromic DOA or Behr phenotype [107,108]). Finally, 89 other non-ataxic autosomal-recessive diseases have been reported, in which the cerebellar ataxia phenotype has occasionally been documented [103].

The worldwide prevalence of ARCAs is estimated to be around 3–6/100,000 individuals [109,110]. Due to the lack of molecular diagnoses, the accessibility of genetic tests, and regional founder effects and consanguinity, this is just an estimation. Among all the ARCAs, FA affects 1:29,000–50,000 people, with the highest prevalence in Caucasians and European countries [109,111,112], and it appears to be the most common, even though the recently described CANVAS is predicted to have a similar frequency [4,100,113].

In the upcoming sections, we delineate the clinical, genetic, and molecular traits of prevalent ARCAs, along with less common mutations which, despite their rarity, exhibit shared pathophysiological traits.

### 3.1. Friedreich’s Ataxia 

Friedreich’s ataxia (FA—OMIM #229300) was first described in the nineteenth century by Nicholaus Friedreich. The onset of clinical symptoms appears before the age of 25 (the mean age of onset is 10–15 years), and disease progression leads to wheelchair dependence within 10 years of disease onset. The classic presentation of FA consists of progressive gait and limb ataxia, dysarthria, polyneuropathy, and sensory loss [114]. Moreover, non-neurological symptoms such as scoliosis (~80% of patients), diabetes (~30%), and hypertrophic cardiomyopathy (>66%) have been reported [115]. Heart failure is, indeed, a common cause of death for patients with FA [102,116]. Differentially to other ARCAs, FA is considered an afferent ataxia because of the degeneration of the large sensory neurons in the dorsal root ganglia (DRG) and posterior column. In addition, the degeneration of the spinocerebellar and corticospinal tracts is well documented, and the lesions in the dentate nucleus cause the major cerebellar phenotype [5,117], while PN dysfunction and cerebral abnormalities are less involved in FA pathophysiology compared to other ARCAs [118,119].

FA is caused by a non-coding GAA trinucleotide expansion within the *FXN* gene encoding frataxin. Most patients (96%) harbor a homozygous GAA triplet expansion (ranging from 40 to 1000 triplets) within the first intron of the *FXN* gene, while the remaining 4% of cases are compound heterozygotes presenting a single allele GAA expansion and a more classical mutation in the other allele [102,116,120]. In contrast with autosomal-dominant cerebellar ataxia, FA is not subject to the anticipation phenomenon and, to date, together with CANVAS, is the only ARCA presenting nucleotide expansion as a disease mechanism. GAA expansion alters the epigenetic pattern (heterochromatin conformation) and/or promotes the formation of RNA/DNA hybrids (R-loops), resulting in a drastic decrease in *FXN* gene transcription and, therefore, reduced levels of frataxin (approximately estimated at 5–30% of the normal levels) [116,121]. Frataxin is a ubiquitously expressed nuclear-encoded mitochondrial protein located in the mitochondrial matrix. Although frataxin has been suggested to be involved in a variety of pathway links to the iron metabolism, the only widely accepted function of frataxin is its essential role as an allosteric regulator of Fe-S clusters’ biogenesis. Since Fe-S clusters are crucial elements in oxidative phosphorylation, iron metabolism, protein translation, and DNA repair/replication, frataxin deficiency leads to low levels of Fe-S cluster biogenesis and, therefore, triggers mitochondrial dysfunction, iron accumulation, oxidative stress, and, most likely, DNA damage accumulation [122,123,124].

### 3.2. CANVAS

Cerebellar ataxia with neuropathy and vestibular areflexia syndrome (CANVAS—OMIM #614575) is a common adult-onset and slowly progressive ataxia disorder characterized by imbalance (80% of patients), sensory neuropathy (almost 100%), bilateral vestibular impairment (60%), chronic cough (>60%), and, occasionally, autonomic dysfunction [125]. The average age of onset has been reported to be 60 years [126]. CANVAS has recently been linked to a biallelic AAGGG repeat expansion in the second intron of the replication factor C subunit 1 (*RFC1)* gene [127]. The allelic carrier frequency of the AAGGG repeat expansion in healthy controls has been documented to be around 0.7%, a percentage similar to healthy carriers of FA suggesting that CANVAS may represent a frequent cause of late-onset ataxia with an estimated prevalence of 1:20,000 individuals [127]. This abnormal expansion has been identified in the poly(A) tail of an AluSx3 element and differs in both size (pathological size ranges from 400 to 2000 repeats) and nucleotide sequence from the reference (AAAAG). Interestingly, the high polymorphism of this locus leads to other expanded non-pathogenic motifs: an AAAAG-extended pentanucleotide whose average size ranges from 15 to 200 repeats and AAAGG repetition ranging from 600 to 1000 [127]. Lately, four novel pathogenic pentanucleotides configurations, both in homozygous and compound heterozygous states with AAGGG, have been associated with CANVAS disease [128]. These expanded regions have been sequenced and registered as AGGGC, AAGGC, AAGAG, and AGAGG, with the extended region always found to be longer than 600 repeats [128]. Alu elements are repetitive (300 bps long) and highly conserved sequences within primate genomes, whose 3′ end has an A-rich region critical for its amplification mechanism. While the pathogenic mechanism of the repeat is unclear, it has been hypothesized that extended AAGGG expansion can induce the inactivation process of the poly(A) tail of the retrotransposon AluSx3 [127]. Furthermore, the existence of a truncating mutation in *trans* with the AAGGG repeat supports the existence of a loss-of-function mechanism [129]. Despite these reports, both the transcript and proteins levels are not affected in patient samples [127,128]. More recently, the formation of G-quadruplexes has been reported to occur with pathogenic motifs, suggesting toxic DNA and toxic RNA modes of pathogenesis or possible R-loops formation [130]. *RFC1* encodes the large subunit of replication factor C, a DNA polymerase accessory protein involved in DNA replication and repair. It is a DNA-dependent ATPase that binds in a structure-specific manner to the 3′ end of a primer hybridized to a template DNA. Therefore, in the presence of ATP, RFC1 activates the DNA polymerase δ and ε to promote the coordinated synthesis of both strands during replication or after DNA damage.

### 3.3. ARSACS

Autosomal-recessive spastic ataxia of Charlevoix-Saguenay (ARSACS—OMIM #270550) is an early-onset cerebellar ataxia initially described in 1979 by Bouchard and colleagues [131]. The disease name is given by the region of Charlevoix-Saguenay-Lac-Saint-Jean (CSLS), in Québec (Canada), where the highest frequency of healthy carriers (1:22 individuals) has been registered [131,132]. Within this population, in 2000, Engert et al., described for the first time two mutations in the *SACS* gene. One of these two mutations, g.8844delT, belongs to a major haplotype shared by 96% of ARSACS families descending from an ancestral founder in the population that settled first in Québec [133]. Nowadays, ARSACS has been diagnosed worldwide, with the identification of more than 200 mutations spread all over the *SACS* gene, and, until recently (before the description of CANVAS), it was considered the second most common recessive ataxia after FA [6]. Most patients are homozygous for *SACS* mutations (45.2%), followed by compound heterozygous (32.9%), and all types of mutation have been reported, with missense substitutions being the most represented; however, macro-deletions, frameshift changes, and nonsense mutations have also been observed [134]. No genotype–phenotype correlation has been documented [135]. ARSACS is clinically characterized by early-onset (the mean for the age of onset is 3 years) and progressive cerebellar ataxia (observed in 78.9% of patients), associated with spasticity (78.1%) and sensory-motor axonal peripheral neuropathy (73.7%) [136]. Its main feature is the progressive degeneration of the cerebellar vermis (remarkable cerebellar atrophy and PN loss) documented by MRIs and post-mortem analyses. Other typical symptoms include dysarthria, distal amyotrophy (likely a consequence of neuropathy), limb weakness, sensory loss, pyramidal signs, and cerebellar eye manifestations (e.g., nystagmus) supported by bilateral demyelination of both corticospinal and dorsal spinocerebellar tracts [131,132,134]. Moreover, ocular coherence tomography analysis has previously detected a peculiar increase in peripapillary retinal nerve fiber layer (RNFL) thickness in ARSACS patients, suggesting it as a putative disease diagnostic tool [137,138]. The *SACS* gene encodes for sacsin, a cytosolic protein highly expressed in the brain (with the highest expression in PNs), skeletal muscle, heart, and dermal fibroblasts [133]. While sacsin’s function is still largely unknown, molecular studies suggest a role of sacsin in protein quality control [139,140], affecting intermediate filament remodeling [141,142,143]. However, the involvement of this protein in alterations in mitochondrial dynamics and quality control, ultrastructure, and bioenergetics is not yet fully clarified [15,143,144].

### 3.4. Ataxia–Telangiectasia

Ataxia–telangiectasia (AT—OMIM #208900) was the first early-onset recessive for which a gene had been identified, and AT is considered a quite common ARCA, accounting for 3–5% of all patients with ARCA. The characteristic clinical symptoms appear between 1 and 4 years of age, including progressive neuronal degeneration (mainly in the cerebellum but also in the cortex and peripheral system), accompanied by oculomotor apraxia (almost 100% of patients), extrapyramidal movements (chorea and dystonia, >80%), immunodeficiency (60–80%), and cancer susceptibility (in particular leukemia and lymphoma, 85%) [145,146]. From a genetic point of view, patients with AT are mainly compound heterozygotes. The vast majority carry nonsense or frameshift mutations over the entire *ATM* gene, resulting in protein loss of function, while the few missense mutations identified affect splicing events [146]. The ATM is a serine/threonine kinase that safeguards genome integrity from double-strand breaks activating more than 20 proteins involved in DNA damage response (e.g., phosphatidylinositol 3-kinase-related protein kinase (PIKK) family, which phosphorylates, in turn, several substrates, like H2AX) and cell cycle regulation (e.g., protein kinase Chk2 and p53). The loss of physiological ATM activity results in an increased radiosensitivity and predisposition to develop cancer due to the missing activation and coordination of repair pathways in response to DNA damage [147,148,149,150].

### 3.5. ARCA2

Autosomal-recessive ataxia type 2 (ARCA2—OMIM #612016), also identified as COQ8A-ataxia, is a rare form of multisystemic ataxia characterized by slowly progressive early onset ataxia, combined with variable symptoms including exercise intolerance (25% of patients), dystonia (28%), epilepsy (32%), and intellectual disability (49%) [151,152,153]. In most cases, disease onset occurs before the age of 6 [154]. ARCA2 is caused by biallelic loss-of-function mutations in *COQ8A* (also known as *ADCK3*), a mitochondrial atypical kinase/ATPase whose biochemical function remains, so far, not completely understood. To date, almost 50 pathogenic variants of different natures (missense, nonsense, frameshift, and deletion) have been identified all along the gene with missense mutations harboring mainly in the COQ8A active site impacting ATPase activity, protein stability, and/or nucleotide binding capacity [154]. COQ8A is located in the matrix face of the inner mitochondrial membrane, where it ensures the integrity of complex Q (enzymes involved in CoQ biosynthesis) and has been suggested to regulate CoQ biosynthesis [155,156,157]. COQ8A deficiency affects the CoQ10 levels in different tissues, resulting in inefficient oxidative phosphorylation, alterations in mitochondrial homeostasis, and oxidative stress due to the lack of antioxidant power [17,158].

## 4. Episodic Ataxias

Episodic ataxia (EA) is a rare group of autosomal-dominant inherited disorders associated with a heterogeneous clinical spectrum manifesting with recurrent cerebellar dysfunction (i.e., spells of truncal ataxia and incoordination) of variable durations and often accompanied by ictal and interictal symptoms [2]. The overall prevalence has been estimated to be less than 1:100,000 individuals, probably underrated because of unidentified causative genes and a lack of genetic tests [9,159]. To date, eight different subtypes have been described, with a recent proposal for two additional ones; however, causative genes have been identified only for five (EA1/*KCNA1*, EA2/*CACNA1A*, EA5/*CACNB4*, EA6/*SLC1A3* and EA8/*UBR4*). Interestingly, four out of the five genes are ion channel proteins involved in excitatory neurotransmission [159]. Clinically, patients show ataxia and vertigo symptoms starting in early childhood [9,159,160]. EA1 (OMIM #160120) is associated with a mutation in *KCNA1* gene encoding the potassium voltage-gated channel α1 subunit Kv1.1, highly expressed in the cerebellum and crucial for the formation of GABAergic synapses on the PNs. Among the 63 *KCNA1* mutations described, most of them are missense mutations leading to a loss-of-function mechanism. The symptoms appear between 2 and 10 years of age, and the attacks can last from seconds to minutes, with varying frequency [9,159]. *CACNA1A* encoding the alpha 1A subunit of the P/Q-type voltage-gated calcium channel (Cav2.1) has been identified to be mutated in EA2 (OMIM #108500). In most of the cases, nonsense and frameshift mutations leading to premature stop codon have been reported [161]. Cav2.1 is highly expressed in PNs and granule cells in the cerebellum conducting synaptic transmission. The age of onset is very early on (2–20 years of age), but, different from EA1, the intermittent spells of ataxia are longer, lasting several hours and, in some cases, even days [9,159].

## 5. X-Linked Degenerative Ataxias

Long CGG repeat expansion in the 5′ non-coding region of the *FMR1* gene are associated with fragile X syndrome (FXS), a common hereditary form of intellectual disability and autism. Interestingly, carriers of relatively short CGG repeat expansions (55–200, premutation range) present distinct inherited degenerative disorders affecting ageing individuals, mainly men. This is the main X-linked ataxia disease identified as fragile X-associated tremor/ataxia syndrome (FXTAS—OMIM #300623) [7,162,163]. Typically, symptoms of FXTAS appear in the fifth decade of life (however, the premutation CGG repeat length has been demonstrated to correlate with the age of onset) [164], accompanied by progressive cerebellar ataxia (85% of all patients), tremor (70%), and cognitive decline (approximately 50%), and are associated with psychiatric manifestations (50%) and peripheral neuropathy (60%) [165]. Interestingly, female carriers of an *FMR1* premutation are associated with an increased risk of primary ovarian insufficiency without developing ataxia and tremors [7,163,166]. From a molecular point of view, in patients with FXTAS, the FMRP (fragile X mental retardation protein) amount is normal, while the *FMR1* mRNA level is drastically elevated in the brain and in leukocytes [167], leading to toxicity (through the production of FRMpolyG by repeat-associated non-AUG (RAN) translation) and causing the cellular injury responsible for the symptoms (intranuclear inclusions and DNA damage through R-loops formation) [163].

## 6. Congenital Ataxias

Congenital cerebellar ataxias are a relatively small group of non-progressive autosomal-recessive ataxias characterized by early-onset cerebellar malformations resulting in motor incoordination and developmental delay, with additional CNS structural defects leading to hypotonia, intellectual disabilities, and apraxia [168,169]. Among the inherited congenital cerebellar ataxias, the most common is Joubert syndrome (OMIM—#213300). It is a clinical and genetic heterogeneous disorder with onset at the neonatal stage and characterized by cerebellum and brainstem malformations (the so-called molar tooth sign), and non-neurological malformations (mainly kidney, retina, and liver) have been reported. The major symptoms of this condition are ataxia, global developmental delay, breathing impairment, and ocular motor apraxia. The overall prevalence is 1:80,000–100,000, and the mutations identified reside in genes encoding for primary cilium proteins, where *INPP5E*, *TMEM216*, *AHI1*, *NPHP1*, *CEP290*, *TMEM67,* and *CC2D2A* are the most frequent mutated genes [170]. Since the primary cilium is an immotile organelle sensing extracellular signals which are transduced within the cell during crucial steps of development and cell function, Joubert syndrome is part of the large family of ciliopathies [170].

## 7. Difficulties in Diagnosis and Implementation of NGS 

To help with the diagnosis, the ERN-RND working groups have proposed flowcharts based on inheritance pattern, prevalence of disease, and specific clinical biomarkers as a guideline (accessed on 10 January 2024 https://www.ern-rnd.eu/wp-content/uploads/2019/08/ERN-RND-Diagnostic-FlowchartAtaxia_FINAL.pdf and accessed on 10 January 2024, https://www.ern-rnd.eu/wp-content/uploads/2020/10/ERN-RND-Diagnostic-Flowchart-for-early-Ataxias_final.pdf). However, a significant number of patients remain undiagnosed, particularly when there is no family history and the manifestation of ataxia occurs later in life. This not only poses a challenge for ataxia-related conditions but also extends to sporadic neurological diseases in general. Specialized centers are increasingly incorporating whole-genome and exome sequencing, often yielding a conclusive diagnosis for sporadic cases. However, it is worth noting that these techniques still face challenges in interpreting results [171,172]. Specifically, the analysis of the entire genome (WGS) for a single individual can yield over 5 million variants, necessitating multiple classification and filtering steps to narrow them down to a few pathogenic candidates. This process must run concurrently with the examination of the patient’s clinical data [173]. For more information, please refer to the ataxia global initiative next-generation sequencing (NGS) working group, dedicated to enhancing methods, platforms, and international standards for the analysis of ataxia using NGS and facilitating the sharing of data (accessed on 10 January 2024 https://ataxia-global-initiative.net/). The ultimate goal is to expand the pool of patients genetically diagnosed with ataxia, making them more accessible for participation in natural history studies and treatment trials. 

## 8. Different Approaches to Ameliorate Disease Outcome

To date, no efficient treatment to halt disease progression has been identified for HA. However, various research fields are actively exploring diverse therapeutic approaches to address these profoundly disabling diseases. In this view, considerable steps forward in understanding pathological mechanisms underlying these diseases have been achieved using pre-clinical disease models, from rodent to human induced pluripotent stem cells (iPSCs).

One of the major challenges remains the low prevalence of these diseases [4,174], making it difficult to recruit patients for clinical trials. Clinical heterogeneity as well as age of onset represent another constraint for the recruitment of patients. Indeed, despite the same genetic mutations or expansions, such as CAG repeats in SCAs, substantial differences are present within the different disease groups [175]. 

This variability justifies the different approaches that are now under investigation to counteract specific disease mechanisms. 

The principal therapeutical strategies developed in cerebellar ataxia (summary in Table 1) pre-clinical and clinical studies are the following (Table 2):

1. Genome editing strategies to correct the pathological mutation involved;
2. Antisense oligonucleotides (ASO) or small RNA structures to interfere with repeat expansion translation or R-loop formation;
3. Gene therapy approaches to rescue the levels of disease-mutated genes or key pathway regulators;
4. Approaches to restore physiological protein levels that are disrupted by altered disease protein homeostasis;
5. Pharmacological treatments, either to target specific pathophysiological mechanisms, reduce toxic metabolites, or supplement crucial compounds.

### 8.1. Genome Editing Strategies to Correct Pathological Mutation

The incredible improvements in genome editing technologies provide interesting perspectives for treating genetic disorders. Indeed, the recent advances in the CRISPR-Cas system offer the possibility to correct disease mutations by introducing a frameshift mutation that disables mutant genes’ function through the excision of large expansion repeats or by base or prime editing [226]. Genome editing has been efficient in reducing the main disease phenotypes and rescuing the molecular defects in several animal models of neurodegenerative disorders (e.g., ALS, HD, AD, PD, and fragile-X syndrome) [227,228,229,230,231]. To date, in the context of cerebellar ataxia, the potentiality of CRISPR-mediated genome editing has been successfully reported mainly in cellular models. The feasibility of deleting the polyQ expansion of *ATXN3* was demonstrated in induced pluripotent stem cells (iPSCs) [187]. The corrected iPSC clones can maintain a normal functionality, such as their pluripotency properties and their neuronal cell differentiation capacity, and the ATXN3-modified protein preserves the ubiquitin-binding ability without the formation of toxic aggregates [187]. Similarly, two different groups successfully proved the efficacy of the targeted excision of different fragments containing the GAA repeats to restore *FXN* mRNA and frataxin protein levels in different iPSC clones [201,202]. However, they showed that deleting exclusively the expanded CAG tract is not always sufficient in reverting pathological FA hallmarks (e.g., axonal spreading and synaptic machinery organization). In fact, it is known that the intronic regions flanking the GAA repeats also contribute to the abnormal DNA methylation on *FXN* promoter, resulting in a transcriptionally non-permissive heterochromatin state [201]. More studies, in particular with in vivo evidence, are necessary to fully understand the feasibility of translating these approaches to the clinic. This is the goal of biotechnology companies that are currently developing and optimizing these strategies. Earlier this year, Prime Medicine announced to have obtained proof-of-concept that the genome-editing removal of expanded GAA repeats [232] corrects *FXN* hypermethylation, restoring normal protein expression (accessed 10 January 2024 https://investors.primemedicine.com/news-releases/news-release-details/prime-medicine-announces-recent-progress-and-highlights-2023). Similarly, CRISPR Therapeutics and Capsida Biotherapeutics are developing a CRISPR/Cas-based genome editing technique for the in vivo treatment of FA and ALS (further information at: accessed 10 January 2024 https://crisprtx.gcs-web.com/news-releases/news-release-details/crispr-therapeutics-and-capsida-biotherapeutics-announce). Moreover, AAV-CRISPR editing and target long-read sequencing have been used in transgenic SCA2 mouse models [183]. Other nucleases have also been used in the cerebellar ataxia field for genome editing. Zinc-finger nucleases (ZFN), a versatile tool for precise genetic modifications, efficiently corrected FA cell phenotypes, such as decreasing aconitase activity and intracellular ATP levels in iPSC-differentiated neurons and cardiomyopathy features in cardiomyocytes [203]. Overall, genome editing technologies for the correction of mutation represent the ideal treatment for polynucleotide expansion ataxia diseases. However, several challenges, including delivery, off-target effects, indel formation by non-homologous end joining, toxicity, and safety concerns, need to be addressed and further investigated to efficiently transfer these technologies to in vivo experimentation.

### 8.2. Antisense Oligonucleotides (ASO) or Small RNA Structures Interfere with Repeat Expansion Translation or R-Loop Formation

A second level on which to tackle the root cause of this type of disease is the mutant mRNA transcript. This can be addressed by taking advantage of (1) antisense oligonucleotides (ASOs), single-stranded oligonucleotides analogues binding pre-mRNA or mRNA affecting protein expression, or (2) RNA interference (RNAi) strategies using double-stranded RNA sequences to decrease mRNA expression. This latter class accounts for small interfering RNAs (siRNAs), short harpin RNAs (shRNAs), and artificial microRNAs (miRNAs). All these molecules bind a specific mRNA target sequence resulting in its degradation or stabilization or preventing the formation of detrimental structures such as R-loops. Experimental results involving RNAi methods have shown promising pre-clinical efficacy in several cerebellar ataxias. The stereotaxic injection of AAV expressing shRNAs or miRNAs targeting *ATXN1* resulted in the significant downregulation of ataxin-1, preventing motor defects and improving molecular and cellular neuropathological phenotypes in different SCA1 mouse models [176,177,178]. Based on the successful outcomes from the rodent models, AAV-ATXN1 miRNAs have been tested in non-human primates. Deep cerebellar nuclei (DCN) injection allowed for the efficient transduction of the cells of interest with a significant reduction in the endogenous *ATXN1* mRNA amount [179].

To maximize mutant gene silencing and most of all preserve wild-type expression and function, an allele-specific shRNA was tested on an SCA3 rat model. Tolerability and the beneficial effects on the neuropathological SCA3-associated symptoms were documented [188]. Moreover, SCA3 mice were also used in the assessment of siRNA and ASOs strategies. siRNAs complementary to *ATXN3* injected in the DCN of SCA3 transgenic mice reached the target and downregulated *ATXN3,* preventing its aggregation [189,190]. More recently, the intracerebroventricular (ICV) delivery of ASOs targeting mutant *ATXN3* successfully restored the potassium channel-mediated PN dysfunction [191] and improved the motor ability of SCA3 mice in addition to a direct correlation with the rescue of specific neurometabolites (choline and, partially, taurine, glutamine, and N-acetyl-aspartate) previously found to be dysregulated [233].

Furthermore, in SCA6, an miRNA targeting the *CACNA1A* IRES, a newly recognized internal ribosomal entry site within the *CACNA1A* C-terminal coding region, reduced ataxia and PN degeneration in a mouse model [196,197]. In SCA7, artificial miRNA complementary to *ATXN7* were injected into the DCN and into the retina of transgenic SCA7 mice. The DCN miRNA-delivery was found to be well tolerated and efficient in improving motor phenotype and PN survival, and, similarly retinal administration did not show any adverse events but achieved satisfactory *ATXN7* downregulation [199,200]. To date, no gene therapy involving siRNA or shRNA has been pursued in clinical trials for HA; however, very encouraging results have been obtained in rodent and non-human primate models [176,179,234].

Another interesting category of gene therapy is represented by ASOs, which have shown remarkable progress in the last decade. These short single-stranded strings of nucleic acids spanning between 8 and 50 nucleotides in length are designed to interfere with RNA via different mechanisms [235]. The first pre-clinical data using ASOs were obtained in a superoxide dismutase 1 (SOD1) ALS rodent model in 2006 [236]. A few years later, it was intrathecally delivered in a clinical trial to patients with SOD1-ALS [237], among which it was well tolerated. The safety and efficacy of the intrathecal administration of ASOs paved the way for the approval by the FDA of Spinraza for the treatment of spinal muscular atrophy (SMA) after a successful phase 3 clinical trial [238]. Encouraging results also come from polyQ diseases such as HD, where Roche, in partnership with Ionis Pharmaceuticals, reported that ASOs safety and tolerance are suitable for further studies [239,240]. Nevertheless, those trials were halted prematurely because of a failure to lower mutant huntingtin [240]. Despite the unfavorable outcomes from some clinical trials, ASOs therapy remains an interesting approach suitable for the treatment of certain polyQ SCAs.

In fact, this strategy was tested also in the ataxia field. Scoles and colleagues screened in vitro 152 ASOs targeting human *ATXN2* and found ASO7 to be a potent and well tolerated molecule resulting in reduced *ATXN2* expression. They proved it in two SCA2 mouse models, corroborating these results with significant improvements in motor performance, restoration of the most dysregulated genes and proteins, and rescue of physiological properties (e.g., PC firing) [184]. Similarly, in FA, the efficacy of ASOs (complementary to the expanded region) in activating the expression of the *FXN* gene and restoring frataxin protein levels near to the physiological levels in different cell lines has been demonstrated. However, the low potency of these compounds could not encourage any pre-clinical study. For this reason, anti-AAG gapmer oligonucleotides, constructs consisting of a central DNA portion flanked by chemically modified RNA with increased binding affinity and efficacy, showed potent results in *FXN* expression activation in patient cell lines, driving its tests in animal models [204,205].

The same genetic modulation can also be addressed in effectors influencing the levels of the disease downstream, into the pathogenetic pathways. This is the case of the nuclear mitogen and stress-activated protein kinase 1 (*MSK1*) that is involved in the phosphorylation activity of a specific amino acid of mutant ataxin-1 [241]. The downregulation of critical components of this pathway leads to reduced levels of mutant ataxin-1, attenuating neurodegeneration and improving disease phenotype in an SCA1 mouse model [241].

### 8.3. Gene Therapy Approaches to Rescue the Levels of Disease-Mutated Genes or Key Pathway Regulators

Gene therapy is a powerful strategy to replace a defective or missing gene often delivered via viral vectors. Significant progresses in the design of new vectors able to cross the BBB, target more efficiently specific cell types, and deliver larger amounts of genetic information open new perspectives in loss-of-function diseases like ARCAs. To date, this approach has been applied only to FA. The first results were obtained by intravenously injecting an AAVrh.10 vector expressing human frataxin (AAVrh.10-CAG-FXN) in mice lacking *FXN* in cardiac and skeletal muscles. The administration not only prevented but also resulted in a complete and rapid recovery of cardiac functionality and the related pathology [206,207,208]. More recently, the post-symptomatic dual intravenous delivery of AAV9-CAG-FXN simultaneously with the intracerebral delivery of AAVrh.10-CAG-FXN resulted in the rapid and full rescuing of FA sensory neuropathy and ganglionopathy [209]. In June 2023, Lexeo Therapeutics completed the first dose cohort in a phase1/2 clinical trial for AAV-based gene therapy for FA cardiomyopathy (NCT05445323). Their study assessed the safety and the tolerability of the treatment. Moreover, cardiac function and biomarkers were evaluated (no reports have been published so far).

### 8.4. Approaches to Restore Physiological Protein Levels That Are Disrupted by Altered Disease Protein Homeostasis

In FA, where protein expression is drastically reduced, a protein replacement strategy using the transactivator of transcription (TAT) peptides has been successfully proven in mice and is now undergoing a clinical trial. The frataxin fusion protein (TAT-FXN) was delivered to different tissues, including heart, DRG, and cerebellum, and to the mitochondria, boosting respiratory chain activity and positively increasing the life span of the conditional heart model with a partial recovery of cardiomyopathy [210]. This strategy is now in a phase 2 clinical trial sponsored by Larimar Therapeutics (NCT05579691) assessing safety, pharmacokinetics, pharmacodynamics, and frataxin levels. Another approach is to activate the transcription of the *FXN* gene with synthetic transcription elongation factor 1 (Syn-TEF1). This was originally assessed in iPSC-derived cardiomyocytes and neurons as well as in mouse xenografts [211], and it was further taken into clinical trials sponsored by Design Therapeutics (NCT05285540).

Moreover, histone deacetylases (HDAC), which are involved in gene expression, are very promising candidates for the treatment of different diseases, from neurodegeneration to cancer therapy [242], and they have been also used for increasing frataxin expression. Several HDAC inhibitors have been tested in pre-clinical studies. For instance, 2-aminobenzamide class I HDAC inhibitors were found to be efficient in promoting frataxin transcription in vitro and in FA mouse models [212]. However, reduced brain penetrability and gastrointestinal toxicity were observed, and, therefore, modified isoforms are currently being optimized for further use in clinical trials. Finally, a class III HDAC inhibitor, nicotinamide (vitamin B3), was demonstrated to have satisfactory bioavailability and safety upregulate frataxin levels in pre-clinical models [213]. The encouraging results that have been collected in pre-clinical in vitro and in vivo models for HA paved the road for a randomized, double-blind, placebo-controlled clinical trial involving patients with SCA3 to test the efficacy of valproic acid (a well-known HDAC inhibitor). In this clinical trial, a significant improvement in the SARA score was reported in the treated patients [192]. Another HDAC3 inhibitor, RG2833, has been tested in clinical trial phase 1 for FA [214], where frataxin deficiency is the main driver of disease pathology. RG2833 noteworthily increases FXN levels; however, it has not been pursued as a metabolite as it was found to be toxic [214].

Another field of thought involves halting the formation of toxic protein aggregates such as toxic polyQ. Despite encouraging results in pre-clinical ataxia models, to our knowledge, no model has yet been considered for clinical trial testing. In SCAs, the synthesis of abnormal polyQ-expanded proteins that often accumulate, forming toxic aggregates, requires an enhanced protein quality control system. To this aim, the expression of molecular chaperones or quality control ubiquitin ligase represent a valid disease-modifying therapy suppressing the disease features of several animal models of SCA1, SCA3, SCA6, and SCA7 [180,243,244,245]. For instance, heat shock protein 90 (Hsp90) inhibitors (e.g., BIIB021) have been shown to be effective in reducing polyQ accumulation in SCA1 [180]. Similarly, targeting the heat-shock response through Hsp90 inhibition (using the KU-32 compound) has been shown to reduce intermediate filament bundles in the fibroblasts of patients with ARSACS, leading to the recovery of mitochondrial membrane potential [218]. However, the absence of evidence regarding the effectiveness of this treatment in pre-clinical animal studies has, thus far, hindered the translation of this approach to the clinical setting. Furthermore, it has been proven that the autophagic/lysosomal pathway is impaired in vulnerable neurons in SCA7 mouse models, but, more interestingly, ATG12 (a gene closely associated with the early phases of autophagy) correlates with disease severity in peripheral blood mononuclear cells (PBMCs) [245].

### 8.5. Pharmacological Treatments to Target Specific Pathomechanisms, Reduce Toxic Metabolites, and Supplement Crucial Compounds

Molecular studies aimed at disclosing the mechanisms involved in cerebellar ataxias have highlighted a converging mitochondrial dysfunction phenotype. Mitochondrial defects can be primarily caused by disease gene mutation or a downstream pathogenic effect. Frataxin deficiency causes decreased ATP production and impaired Fe-S cluster assembly, resulting in ROS production and oxidative stress. To counteract oxidative stress, antioxidant molecules are now under investigation in FA pre-clinical and clinical trials, including Omaveloxolone (Omav), which has been recently approved by the FDA as the first approved FA treatment [246]. Other antioxidant-targeting medications have also showed beneficial effects in FA and are currently being tested in clinical trials (i.e., Vatiquinone). Controversial results were collected by administrating coenzyme Q10 (CoQ10) and Idebenone, a CoQ10 analogue scavenging inner mitochondrial membrane ROS, already undergoing a clinical trial, although without any fully established benefits in patients with FA [247]. However, a diet supplemented with coenzyme Q10 (CoQ10) has been shown to significantly recover motor coordination in SCA3 mice, reducing PN loss and skeletal muscle atrophy, where CoQ10 efficacy was explained by the improved autophagy-mediated clearance of mutant ataxin 3 [193]. Chronic CoQ10 oral supplementation induces a mild motor improvement in patients with ARCA2 [223], and in vitro studies correlated CoQ10 administration with the restoration of mitochondrial dysfunction and calcium dysregulation [17]. It is important to note that, in the context of ARCA2, the use of CoQ10 and its analogues for therapeutical purposes have different meanings. In fact, Coq8a depletion directly impacts CoQ10 biosynthesis, so exogenous supplementation can compensate the defective levels of this molecule, crucial for several physiological processes (e.g., electron chain transport, antioxidant power, regulation of cytoplasm pH, membrane fluidity, etc.).

MitoQ, an antioxidant ubiquinone which specifically accumulates within the mitochondria, has been proposed to be a promising therapeutic drug for several neurodegenerative diseases, including ataxias. MitoQ has been described to prevent cell death in FA fibroblasts [217]. Moreover, pre- and post-symptomatic in vivo MitoQ administration restores mitochondrial morphology and function and positively improves motor coordination and gait imbalance in an SCA1 mouse model, attenuating PN degeneration [19]. Recently, MitoQ chronic treatment in ARSACS mice displayed beneficial effects in PN survival and DCN innervation, preventing the motor decline associated with sacsin depletion [219].

Nicotinamide riboside supplementation has been found to be efficient in mitigating the AT phenotype. This treatment boosts the NAD^+^ levels and, by promoting mitophagy, prevents senescence and neuroinflammation, finally resulting in attenuated PN loss and other neuropathological AT features such as dsDNA damage and mitochondrial dysfunction, followed by improved motor ability in *Atm^−/−^* mice [221].

Several studies have reported changes in PN’s intrinsic excitability and impairment in synaptic signaling pathways, preceding neurodegeneration in numerous cerebellar ataxia mouse models, suggesting the restoration of electrophysiological properties, both via genetic and pharmacological approaches, as an attractive therapeutical strategy for cerebellar ataxias. Potassium channels, which are key regulators of PN excitability and dendritic plasticity, are attractive targets. The FDA-approved drug 4-aminopyridine (4-AP) ameliorates motor coordination and restore PN firing in SCA1 and SCA6 mice [181,198]. PN activity is also under the fine control of the small conductance calcium-activated potassium channel (SK), and oral administration of a positive modulator of the SK2/3 channels (NS13001 and chlorzoxazone) has provided significant improvements in motor deficits and neuropathological manifestation in SCA2 mice [185,186].

Finally, compounds acting on the ion channel and glutamate transporters effectively improved ataxic phenotypes. Ceftriaxone administration in SCA28 and ARSACS murine models has shown significant improvements in motor defects, mitigating PN loss by restoring calcium homeostasis and reducing neuroinflammation [143,220]. The extreme rarity of patients with SCA28 has complicated the transfer of Ceftriaxone treatment to clinics in the context of ataxia. However, with recent and promising results demonstrating the efficacy of this medication in an ARSACS mouse model, there is potential for facilitating the establishment of a clinical study involving various patients with ataxia. Regardless of the intrinsic differences among HAs, there are common pathological pathways that are shared not only between HAs but also with other neurodegenerative diseases, which makes it appealing to retest the same pharmacological approach among other diseases (drug repurposing). For instance, Riluzole, a glutamate release inhibitor and activator of calcium-activated potassium channels, one of the few drugs approved by the FDA for ALS treatment, has been tested in clinical trial for SCA2. The ATRIL study was a randomized, double-blind, placebo controlled, multicenter clinical trial study that was conducted on 45 patients with SCA2 [248] but, unfortunately, did not show any benefit in the improvement of motor deficits, probably because the recruited patients had marked PN atrophies but also because these modulators have a poor capacity for bypassing the BBB. However, previous phase 2 clinical trials in mixed cohorts of patients with different cerebellar ataxias (SCA1, 2, 17, 28, and FA) reported encouraging results after Riluzole administration [224,249], indicating that further investigation are needed to prove the efficacy of this compound in ataxia treatment. Currently, Biohaven Pharmaceutical, Inc. has a pro drug of Riluzole, named Troriluzole, in a phase 3 clinical trial (NCT03701399) for different spinocerebellar ataxias [225], acting on ameliorating PN functionality.

Another molecule that is commonly used to halt nicotine addiction is Varenicline, a partial agonist of α4β2 nicotinic acetylcholine receptors, which was tested in a double-blind, placebo-controlled trial among patients with SCA3, showing a marked improvement in the Scale for the Assessment and Rating of Ataxia (SARA) scores compared to the placebo control group [194]. An additional molecule acting on receptors that control neuronal excitability that has been tested in a randomized double-blind placebo-controlled trial of 57 patients with FA and multiple system atrophy-cerebellar subtypes (MSA-C) is Amantadine, a noncompetitive N-methyl-D-aspartate (NMDA) agonist which has been shown to be effective for the treatment of Parkinson’s disease [222]. NMDA showed positive outcomes for patients with MSA-C but not for patients with FA. Interestingly, positive results of the Amantadine treatment also came from a non-randomized open-label study (NCT00950196) that involved ataxia–telangiectasia [250].

Another interesting compound that was tested in an open-label trial in FA and gave some promising results in pre-clinical models of SCA3 is resveratrol, acting on sirtuin 1 (SIRT1), despite the fact that its underlying mechanisms are not yet understood [195]. Regarding FA, the resveratrol open-label trial did not show improvement in the FXN level; nevertheless, a positive clinical effect might exist with a high dosage. Further assessment in a randomized placebo-controlled trial is needed to assess the beneficial effect of this compound [251]. However, the results of the phase 2 study presented at the International Congress for Ataxia Research in November 2022 were reported to be negative. Interesting technology has been developed by Erydel, which is able to deliver intra-erythrocyte dexamethasone for ataxia–telangiectasia (NCT02770807). In fact, a short-term trial was the first pioneer in this sense, testing the administration of oral betamethasone, which showed corticosteroid side effects; therefore, to overcome this, issue autologous erythrocytes were loaded with dexamethasone phosphate (DPS) (Erydel). Like betamethasone, dexamethasone possesses a significant anti-inflammatory potency and does not exhibit mineralocorticoid activity, and erythrocyte phosphatases convert DSP to dexamethasone, which is then released into the bloodstream [252]. Moreover, a case report study reported the positive effect of oral administration of dexamethasone in a patient with ataxia–telangiectasia, where an improvement of 7 points in the patient’s SARA score was observed over the 28 days of the treatment [253]. This represent a huge improvement that is worth further investigations.

Autophagy impairment seems to underlie or at least contribute to disease pathogenesis in different NDDs. In this context, lithium, known to induce autophagy and aid in the clearance of mutant huntingtin as well as alpha-synuclein [254], was used to treat SCA1 in a pre-clinical mouse model, resulting in improved motor coordination without an increase in lifespan [182]. However, the same effect was not seen in a SCA3 mouse model nor in a double-blind, placebo-controlled phase 2 clinical trial for SCA3. The treatment, involving 63 patients, was tolerated, but no beneficial effect was observed in the patients’ Neurological Examination Score for Spinocerebellar Ataxia (NESSCA) [255]. Similarly, when tested on a small cohort of patients with SCA2, no significant improvement was reported following treatment in their SARA score [256]. Despite the unfavorable outcomes in clinical trials, it remains crucial to report these results, as they could provide valuable insights for future therapeutic approaches and new clinical trials’ design.

## 9. Ataxia FDA-Approved Drugs and Treatments

As of today, no resolutive treatment is available to treat HA. Most interventions rely on palliative care to treat symptoms. Multiple drugs have so far been inconclusive in the treatment of ataxia and further studies are needed to assess the safety and the efficacy of the previously described pharmacological treatments (Riluzole, Varenicline, lithium, etc.). Vitamins and anti-oxidant nutrient supplements, for instance, Vitamin E, coenzyme Q, and resveratrol, are often recommended and have been shown to improve some aspect of the pathology, in particular in ataxias which involve main mitochondria dysfunctions like FA [257], although the data collected are still insufficient to support the use of mitochondrial enhancement in patients with ataxia. Other approaches involve the application of small magnetic fields to modify cerebellar activity using transcranial magnetic stimulation (TMS), although major trials are needed to support the safety and outcomes of long-term treatment with these kind of stimulations [258]. These techniques have already been approved by the FDA for the treatment of major depression and certain types of migraines [225]. Importantly, patients are encouraged to follow physiotherapy and physical exercising, which can often improve quality of life and independence while coping with a long-lasting disease [225,259].

Finally, in early 2023, the FDA approved the first therapeutic agent for the treatment of patients with FA who are older than 16 years, i.e., Omaveloxolone (SKYCLARYS™). Omaveloxolone is a nuclear factor erythroid 2-related factor 2 (Nrf2) activator, which helps in improving mitochondrial function, restoring redox balance, and reducing inflammation. Patient with FA that participated in the MOXIe (NCT02255435) double-blind, randomized, placebo-controlled, parallel-group phase 2 trial that determined the safety and efficacy of Omaveloxolone over 48 weeks reported significant improvements in their Modified Friedreich’s Ataxia Rating Scale (mFARS) scores compared to the placebo-controlled group [215,216]. The approval of this drug after a certain number of failed clinical trials that did not reach their primary endpoints for FA [260] gives hope to many other forms of HA and represents a milestone in the treatment of ataxias.

## 10. Main Challenges and Limiting Factors

One of the main challenges in developing efficient clinical treatments is the prevalence of HAs, as majority of these conditions are in the range of 1–3/100,000 [4]. Another important constraint is the heterogeneity between the different forms of ataxia, even when considering same genetic conditions, such as CAG repeats in SCAs. The brain structures that are affected can be quite different, which reflects the broad spectrum of symptoms which patients experience: for example, SCA7 mainly involves retinal degeneration, SCA6 presents relatively pure cerebellar involvement, while other forms present cerebellar and brainstem dysfunctions [175,261].

Another important feature to take into account is the variability in genetic expansion that characterizes some forms of HA, where different sizes in repeats’ expansion are often associated with different symptomatology severities and disease onset [25,262]. In addition, different forms of HA can vary among geographical and ethnical groups [174], information which needs to be considered for patients’ recruitment into clinical trials. Importantly, the diagnosis of patients might take some time if no clear genetic cause is known, and the lack of efficient pre-symptomatic biomarkers in familial forms, such as fluidic biomarkers which can be measured in cerebrospinal fluid (CSF) or in the blood of patients as indicators of disease progression, makes it difficult to intervene before disease onset or to monitor the efficacy of a treatment.

Challenges are also found in developing the appropriate pre-clinical models. Indeed, as discussed, many promising pre-clinical results have not been translated into clinic settings. Being able to develop humanized rodent models or human-based models, such as iPSC, is the current challenge. While the generation of induced cortical neuron and organoids has been achieved without major challenges [11,263,264,265,266,267], the generation of PN and cerebellar organoids needs further optimization as the protocols available present low purity and maturity, and the survival of PN in cultures is still challenging [268,269,270,271]. These represent powerful tools for drug testing and the study of the pathophysiological mechanisms underlying HAs, allowing researchers to investigate pathogenic variants while maintaining the genetic background of patients. Moreover, the wide access to CRISPR-Cas genome editing allows researchers to generate isogenic controls from the same patient cell line [272], pointing toward the concept of personalized medicine.

Despite all the progress that has been made in a pre-clinical set-up, where a variety of molecules and gene therapy approaches have showed promising results, the step toward clinical trial and drug approval is challenging. Finally, innovation in clinical trial design will likely play a role in the future. For instance comparing multiple treated groups to a single placebo arm or incorporating cross-over arms can reduce the number of recruited patients and make trials more appealing to participants [273].

## 11. Conclusions

Nowadays, discovering an effective remedy for neurodegenerative diseases continues to be a significant challenge in the field of medicine, and this challenge is anticipated to persist for the coming decades. Despite notable advancements in both basic and clinical research, there is still a lack of understanding regarding the pathophysiological processes that could be targeted by therapy.

Regarding HAs, the rarity of certain forms coupled with the inherent heterogeneity in terms of genetics and clinical manifestation pose a challenge in efficiently targeting these rare forms of disease and identifying common pathways. The utilization of pre-clinical models, which are often scarce, remains one of the most valuable tools to unravel the mechanistic pathophysiology, contributing to the development of targeted and, therefore, more effective therapies.

Finally, it is crucial to consolidate efforts from a clinical perspective, emphasizing the significance of natural history studies. These studies play a pivotal role in identifying appropriate criteria for the design of clinical trials, exemplified by the recently approved treatment of Omaveloxolone for FA. In this case, the approval process hinged on the insights gained from natural history studies and the effective development of pre-clinical models to pinpoint specific pathological targets.

## Figures and Tables

**Table 1:** Summary of HA features.

Disease	OMIM ID	Location	Gene/Locus	Inheritance	Clinical Features and Phenotype
Spinocerebellar ataxia type 1 (SCA1)	#164400	6p22.3	*ATXN1*	Autosomal-dominant	Cerebellar ataxia (characterized by Purkinje neuron and dentate nucleus neuron loss) with variable involvement of the brainstem and spinal cord. Cognitive decline has been also observed
Spinocerebellar ataxia type 2 (SCA2)	#183090	12q24.12	*ATXN2*	Autosomal-dominant	Cerebellar ataxia (characterized by olivopontocerebellar degeneration) associated with oculomotor defects, parkinsonism, cognitive impairment, and peripheral neuropathy
Spinocerebellar ataxia type 3 or Machado–Joseph Disease (SCA3 or MJD)	#109150	14q32.12	*ATXN3*	Autosomal-dominant	Cerebellar ataxia (characterized by the degeneration of deep cerebellar nuclei neurons) with pyramidal and extrapyramidal signs. In some cases, peripheral neuropathy and parkinsonism have been also detected
Spinocerebellar ataxia type 6 (SCA6)	#183086	19q13.13	*CACNA1A*	Autosomal-dominant	Pure cerebellar ataxia characterized by Purkinje neuron degeneration. In a few cases, basal ganglia dysfunction has been reported
Spinocerebellar ataxia type 7 (SCA7)	#164500	3p14.1	*ATXN7*	Autosomal-dominant	Cerebellar ataxia, dysarthria, spasticity, and peculiarly retinal degeneration
Spinocerebellar ataxia type 17 (SCA17)	#607136	6q27	*TBP*	Autosomal-dominant	Cerebellar ataxia (characterized by Purkinje neuron loss) associated with the degeneration of different other brain region (cerebral cortex, basal ganglia, and cingulate and hippocampal gyri) resulting in pyramidal signs, psychiatric dysfunction, and dementia
Spinocerebellar ataxia type 27B or GAA-FGF17 ataxia (SCA27B)	#620174	13q33.1	*FGF14*	Autosomal-dominant	Cerebellar ataxia (characterized by Purkinje neuron loss, in particular in the vermis) in combination with nystagmus, dysarthria, vertigo, spasticity, or peripheral axonal neuropathy
Friedreich’s ataxia (FA)	#229300	9q21.11	*FXN*	Autosomal-recessive	Progressive gait and limb ataxia, dysarthria, polyneuropathy, and sensory loss due to the dysfunction of the spinocerebellar and pyramidal tracts and the dorsal column. Cerebellum is barely affected, with only dentate nucleus neuron loss
Cerebellar ataxia with neuropathy and vestibular areflexia syndrome (CANVAS)	#614575	4p14	*RFC1*	Autosomal-recessive	Cerebellar ataxia (characterized by imbalance) associated with bilateral vestibulopathy, sensory neuropathy, and, occasionally, autonomic dysfunction and chronic cough
Autosomal-recessive spastic ataxia of Charlevoix-Saguenay (ARSACS)	#270550	13q12.12	*SACS*	Autosomal-recessive	Cerebellar ataxia (characterized by Purkinje neuron degeneration) later associated with pyramidal tract signs, spasticity, and peripheral neuropathy
Ataxia–telangiectasia (AT)	#208900	11q22.3	*ATM*	Autosomal-recessive	Cerebellar ataxia and progressive degeneration of different neuronal populations associated with oculomotor apraxia, extrapyramidal movements, and immunodeficiency
Autosomal-recessive ataxia type 2 (ARCA2)	#612016	1q42.13	*ADCK3*	Autosomal-recessive	Cerebellar ataxia combined with exercise intolerance, dystonia, epilepsy, and intellectual disability
Episodic ataxia 1 (EA1)	#160120	12p13.32	*KCNA1*	Autosomal-dominant	Periodic short ataxia and myokymia attacks
Episodic ataxia 2 (EA2)	#108500	19qp13.13	*CACNA1A*	Autosomal-dominant	Long intermittent ataxic events
Fragile X-associated tremor/ataxia syndrome (FXTAS)	#300623	Xq27.3	*FMR1*	X-linked dominant	Cerebellar ataxia accompanied by tremor, peripheral neuropathy, cognitive decline, and psychiatric signs
Joubert syndrome	#213300	9q34.3	*INPP5E*	Autosomal-recessive	Ataxia (caused by cerebellum and brainstem malformations), ocular motor apraxia, and non-neurological signs such as breathing impairment, kidney, and liver alterations

**Table 2:** Summary of different treatment strategy and outcomes for HA.

Strategy		Stage	Model	Outcomes	References
**Spinocerebellar ataxia type 1 (SCA1)**					
Small RNA structures	shRNA and miRNA	Pre-clinical	SCA1 mouse models	Improvement in motor coordination, restoration of cerebellar morphology, and absence of ataxin-1 inclusions	[176,177,178]
Small RNA structures	miRNA	Pre-clinical	SCA1 non-human primate	Reduction in endogenous *ATXN1* mRNA	[179]
Proteostasis	HSP inhibitor (BIIB021)	Pre-clinical	Human SCA1 cell lines	Reduction in ataxin-1 aggregates	[180]
Pharmacological treatment	MitoQ	Pre-clinical	SCA1 mouse models	Restoration of mitochondrial function, attenuation of PN degeneration, and improvement in motor coordination	[19]
Pharmacological treatment	4-animopyridine	Pre-clinical	SCA1 mouse model	Restoration of PN firing, improvement in motor coordination, and partial protection against cell atrophy	[181]
Pharmacological treatment	Lithium	Pre-clinical	SCA1 mouse model	Improvement in motor coordination	[182]
**Spinocerebellar ataxia type 2 (SCA2)**					
Genome editing	CRISPR	Pre-clinical	SCA2 mouse models	CAG repeat size and rearrangements and frequency of integration	[183]
Small RNA structure	ASO	Pre-clinical	SCA2 mouse models	Improvement in motor performance and restoration of physiological properties and deregulated genes and proteins	[184]
Pharmacological treatment	Chlorzoxazone	Pre-clinical	SCA2 mouse model	Normalization of PN firing and alleviation of ataxic phenotype	[185]
Pharmacological treatment	NS13001	Pre-clinical	SCA2 mouse model	Improvement in motor ability and reduced PN degeneration	[186]
**Spinocerebellar ataxia type 3 (SCA3)**					
Genome editing	CRISPR	Pre-clinical	Human iPSCs	Restoration of ataxin-3 functionality without the formation of toxic aggregates	[187]
Small RNA structure	shRNA	Pre-clinical	SCA3 rat model	Reduced ataxin-3 inclusions and prevention of neurodegeneration	[188]
Small RNA structure	siRNA	Pre-clinical	SCA3 mouse model	*ATXN3* downregulation and prevention of its aggregation	[189,190]
Small RNA structure	ASO	Pre-clinical	SCA3 mouse model	Improvement in motor ability, restoration of PN dysfunction, and rescue of altered neurometabolites	[191]
Gene activation	HDAC inhibitor, valproic acid	Clinical phase ½	SCA3 patients	Patients treated with valproic acid improved their locomotor function (SARA scale)	[192]
Pharmacological treatment	Coenzyme Q10	Pre-clinical	SCA3 mouse model	Recovery of motor coordination, reduced PN degeneration, and muscle atrophy	[193]
**Pharmacological treatment**	Varenicline	Clinical	SCA3 patients	Improvement in SARA scale score	[194]
Pharmacological treatment	Resveratrol	Clinical	SCA3 mouse model	Reduced motor incoordination	[195]
**Spinocerebellar ataxia type 6 (SCA6)**					
Small RNA structure	miRNA	Pre-clinical	SCA6 mouse model	Reduced ataxic phenotype and PN degeneration	[196,197]
Pharmacological treatment	4-aminopyridine	Pre-clinical	SCA6 mouse model	Improvement in motor ability and restoration of PN firing	[198]
**Spinocerebellar ataxia type 7 (SCA7)**					
Small RNA structure	miRNA	Pre-clinical	SCA7 mouse model	Improvement in motor deficit, increased PN survival, and *ATXN7* downregulation	[199,200]
**Spinocerebellar ataxia type 28 (SCA28)**					
Pharmacological treatment	Ceftriaxone	Pre-clinical	SCA28 mouse model	Reduced PN degeneration and improvement in motor performance	[143]
**Friedreich’s Ataxia (FA)**					
Genome editing	CRISPR	Pre-clinical	Human iPSCs	Deletion of the expanded CAG tract is not always sufficient to revert the phenotype	[201,202]
Genome editing	ZFN	Pre-clinical	Human iPSC-derived neurons and cardiomyocytes	Decreased aconitase activity and ATP levels in iPSC-derived neurons and corrected cardiomyopathy in cardiomyocytes	[203]
Small RNA structure	ASO	Pre-clinical	Human FA cell lines	Activation of *FXN* expression and consequent restoration of frataxin levels	[204]
Small RNA structure	Gapmer	Pre-clinical	Human FA cell lines	Activation of *FXN* expression	[205]
Gene therapy	AAVrh10-CAG-hFXN	Pre-clinical	FA mouse model	Complete and rapid recovery of cardiac functionality	[206,207,208]
Gene therapy	AAV9-CAG-hFXN-HA, AAVrh.10-CAG-hFXN-HA	Pre-clinical	FA mouse model	Complete and rapid rescue of sensory neuropathy and ganglionopathy	[209]
Gene therapy	LX2006 (AAVrh.10hFXN)	Clinical phase 1/2	Patients with FA	Ongoing, sponsored by Lexeo Therapeutics, New York, NY, USA (NCT05445323)	
Protein replacement	TAT peptides	Pre-clinical	FA mouse model	Decreased neurite degeneration and apoptotic markers resulting in increased cell survival and restoration of mitochondrial features	[210]
Protein replacement	TAT peptides	Clinical phase 2	Patients with FA	Ongoing, sponsored by Larimar Therapeutics, Bala Cynwyd, PA, USA (NCT05579691)	
Gene activation	Syn-TEF1	Pre-clinical	Human iPSC-derived neurons and cardiomyocytes	Activation of *FXN* expression	[211]
Gene activation	Syn-TEF1	Clinical phase 1a	Patients with FA	Ongoing, sponsored by Design Therapeutics, Carlsbad, CA, USA (NCT05285540)	
Gene activation	HDAC inhibitors, 2-aminobenzamide	Pre-clinical	Human cell lines and FA mouse models	Activation of *FXN* expression	[212]
Gene activation	HDAC inhibitors, nicotinamide	Pre-clinical	FA mouse model	*FXN* upregulation	[213]
Gene activation	HDAC inhibitors, RG2833	Clinical phase 1	Patients with FA	Increased *FXN* levels, but a toxic metabolite was detected	[214]
Pharmacological treatment	Omaveloxolone	Clinical phase 2	Patients with FA	Approved, sponsored by Biogen, Cambridge, MA, USA (NCT02255435)	[215,216]
Pharmacological treatment	Vatiquinone	Clinical phase 2/3	Patients with FA	Ongoing, sponsored by PTC Therapeutics, South Plainfield, NJ, USA (NCT04577352)	
Pharmacological treatment	MitoQ	Pre-clinical	FA cell lines	Reduced cell death	[217]
**Autosomal-Recessive Spastic Ataxia of Charlevoix-Saguenay (ARSACS)**					
Proteostasis	HSP inhibitor (KU-32)	Pre-clinical	Human ARSACS cell lines	Reduction in vimentin bundles and restoration of mitochondrial membrane potential	[218]
Pharmacological treatment	MitoQ	Pre-clinical	ARSACS mouse model	Decreased PN degeneration, increased DCN innervation, and prevention of motor decline	[219]
Pharmacological treatment	Ceftriaxone	Pre-clinical	ARSACS mouse model	Restoration of calcium homeostasis, reduced neuroinflammation, and improvement in motor ability	[220]
**Ataxia–telangiectasia (AT)**					
Pharmacological treatment	Nicotinamide riboside	Pre-clinical	AT mouse model	Prevention of neuroinflammation, reduced mitochondrial dysfunction and PN death, and improvement in motor ability	[221]
Pharmacological treatment	Amantadine	Clinical phase 4	Patients with AT	Improvement in ataxic phenotype, involuntary movements, and parkinsonism symptoms	[222]
Pharmacological treatment	Dexamethasone	Clinical phase 3	Patients with AT	Sponsored by Erydel, South San Francisco, CA, USA(NCT02770807)	
**Autosomal-Recessive Ataxia tye-2 (ARCA2)**					
Pharmacological treatment	Coenzyme Q10	Clinical	Patients with ARCA2	Mild improvement in motor features	[223]
**Multi-disease trials**					
Pharmacological treatment	Riluzole	Clinical phase 2	Patients with SCA1, SCA2, SCA17, SCA28 and FA	Improvement in ICARS scale score	[224]
Pharmacological treatment	Riluzole	Clinical phase 3	Patients with SCA1, SCA2, SCA3, SCA6, SCA7, SCA8 and SCA10	Ongoing, sponsored by Biohaven Pharmaceutical, Inc., New Haven, CT, USA (NCT03701399)	[225]
